# Oral manifestations of COVID‐19 and its management in pediatric patients: A systematic review and practical guideline

**DOI:** 10.1002/cre2.776

**Published:** 2023-08-21

**Authors:** Kamyar Nasiri, Sahar Tehrani, Meysam Mohammadikhah, Morteza Banakar, Mojgan Alaeddini, Shahroo Etemad‐Moghadam, Gustavo V. O. Fernandes, Artak Heboyan, Shima Imannezhad, Farid Abbasi

**Affiliations:** ^1^ Department of Dentistry Islamic Azad University Tehran Iran; ^2^ Department of Pediatric Dentistry, School of Dentistry Ahvaz Jundishapour University of Medical Sciences Ahvaz Iran; ^3^ Department of Oral and Maxillofacial Surgery School of Dentistry, Alborz University of Medical Sciences Karaj Iran; ^4^ Dental Research Center, Dentistry Research Institute Tehran University of Medical Sciences Tehran Iran; ^5^ Department of Pediatric Dentistry, Faculty of Dentistry Shahed University Tehran Iran; ^6^ Department of Periodontics and Oral Medicine University of Michigan School of Dentistry Ann Arbor Michigan USA; ^7^ Centre for Interdisciplinary Research in Health (CIIS) Universidade Católica Portuguesa Viseu Portugal; ^8^ Department of Prosthodontics, Faculty of Stomatology Yerevan State Medical University after Mkhitar Heratsi Armenia; ^9^ Department of Pediatrics, Faculty of Medicine Mashhad University of Medical Sciences Mashhad Iran; ^10^ Department of Oral Medicine, Faculty of Dentistry Shahed University Tehran Iran

**Keywords:** child, COVID‐19, oral health, oral manifestations, oral mucosal lesions, pediatric patients

## Abstract

**Objectives:**

The severe acute respiratory syndrome coronavirus 2 (SARS‐CoV‐2) virus causes coronavirus disease 2019 (COVID‐19), a respiratory infection that has spread worldwide and is responsible for a high death toll. Although respiratory symptoms are the most common, there is growing evidence that oral signs of COVID‐19 can also be seen in children. The purpose of this systematic review is to provide a comprehensive analysis of the available data on the oral manifestations of COVID‐19 in children and to recommend appropriate methods of diagnosis and treatment.

**Methods:**

A systematic search of the MEDLINE, EMBASE, Scopus, and Web of Science databases was done to discover relevant papers published between their establishment and January 2023. Articles detailing oral symptoms in pediatric patients with confirmed COVID‐19 infection were included, and data on clinical characteristics, diagnosis, treatment, and outcomes were extracted and evaluated.

**Results:**

A total of 24 studies involving 2112 pediatric patients with COVID‐19 were included in the review. The most common presentations are oral lesions, taste and smell disorders, oral candidiasis, hemorrhagic crust, tongue discoloration, lip and tongue fissuring, gingivitis, and salivary gland inflammation. These manifestations were sometimes associated with multi‐system inflammatory syndrome in children (MIS‐C) or Kawasaki disease (KD). Management strategies varied depending on the severity of the oral manifestation and ranged from symptomatic relief with topical analgesics to systemic medications.

**Conclusion:**

Oral symptoms of COVID‐19 are relatively prevalent in juvenile patients and can be accompanied by severe systemic diseases, such as MIS‐C or Kawasaki illness. Early detection and adequate care of these oral symptoms are critical for the best patient results. Understanding the underlying pathophysiology and developing targeted treatments requires more investigation.

## 1 INTRODUCTION

The coronavirus disease 2019 (COVID‐19) pandemic has greatly impacted the world, with millions of recorded cases and fatalities. The global community has struggled with its influence on public health, social and economic institutions, and daily living (Ciotti et al., 2020). Over 700 million cases of COVID‐19 have been confirmed worldwide as of March 2023, with an estimated 6.8 million fatalities, according to the World Health Organization (WHO) (WHO, 2023). It has impacted people of all ages worldwide; however, the disease's prevalence and severity change with age. The WHO reports that adults, rather than children, contribute to most COVID‐19 cases worldwide (WHO, 2023). Concern about the prevalence and potential consequences of COVID‐19 in pediatric patients has grown in recent years. COVID‐19 was shown to pose a threat to children of all ages and sexes (Delahoy, 2021; Dong et al., 2020). COVID‐19 is caused by the severe acute respiratory syndrome coronavirus 2 (SARS‐CoV‐2) virus, which mostly affects the respiratory system and produces symptoms such as cough, fever, and shortness of breath. As the pandemic has advanced, it has become clear that COVID‐19 may also impact other systems and organs, including the oral cavity, which is responsible for vital processes like chewing, swallowing, and speaking. A tiny number of children infected with COVID‐19 have acquired a disorder known as multi‐system inflammatory syndrome in children, despite the fact that most children infected with COVID‐19 exhibit minimal or no symptoms (MIS‐C). As the name implies, MIS‐C affects many organ systems, including the oral cavity, and in certain instances, hospitalization is necessary (Panigrahy et al., 2020; Vogel et al., 2021).

The mouth cavity is a portal to the respiratory and digestive systems and can serve as a location of entrance and transmission for a variety of infections, including SARS‐CoV‐2 (Banakar et al., 2020; MohammadSadeghi et al., 2020). Studies have shown that COVID‐19 can cause various oral manifestations in adult and pediatric patients. These include symptoms such as oral ulcers, dry mouth, taste disturbances, and gingivitis. The incidence and severity of these manifestations vary, and the exact mechanism of occurrence is not yet fully understood. Oral manifestations can cause pain and discomfort and affect children's quality of life (Kusiak et al., 2021; Tuter, 2022). Understanding the oral signs of COVID‐19 in pediatric patients is vital, as it can facilitate the early identification and treatment of the condition. Due to age‐related changes in immunological responses and the limited availability of effective therapies, managing COVID‐19 in pediatric patients can be complicated. In addition, oral symptoms might complicate the management of COVID‐19 in young patients, as it can interfere with their ability to eat, drink, and take medicines. Therefore, it is necessary to develop effective oral manifestations of COVID‐19 management options for pediatric patients (Bhujel et al., 2021; Halepas et al., 2021; Samuel et al., 2021).

Given the increased frequency of COVID‐19 in pediatric patients and the potential impact of oral manifestations on their treatment and clinical outcomes, it is crucial to have a comprehensive understanding of these manifestations' clinical features and management (American Academics of Pediatrics, 2023; CDCP, 2023; Payne et al., 2021; Santos et al., 2022). In light of this, this systematic review aims to provide a comprehensive summary of the existing information about the oral symptoms of COVID‐19 in pediatric patients. In addition, we intend to identify knowledge gaps and suggest future research subjects that might help create evidence‐based guidelines for diagnosing and treating these symptoms in pediatric patients. In addition, it can aid medical professionals in recognizing the earliest signs of COVID‐19 in pediatric patients, leading to earlier treatment and improved clinical results.

## 2 METHODS

### 2.1 Study design

This systematic review was conducted according to the Preferred Reporting Items for Systematic Reviews and Meta‐Analyses (PRISMA) guidelines (Page et al., 2021). A systematic review protocol was developed before conducting the study, including the research question, inclusion and exclusion criteria, search strategy, the data extraction process, and the data analysis plan.

### 2.2 Eligibility criteria

The population‐intervention‐comparison‐outcome (PICOS) approach addressed the following question: “In pediatric patients with COVID‐19, what are the characteristics of oral manifestations, compared with pediatric patients with COVID‐19 without oral manifestations, and what are the associated outcomes and management strategies”. In this process, the population (P) was pediatric patients (0–18 years) with confirmed COVID‐19. Intervention (I) was identifying, diagnosing, and managing oral manifestations related to COVID‐19. The comparison (C) was pediatric patients with COVID‐19 without oral manifestations or those with oral manifestations unrelated to COVID‐19. The primary outcomes (O) evaluated were the type and severity of oral manifestations in pediatric patients with COVID‐19; intervention strategies for managing oral manifestations; duration of oral manifestations; any associated complications or adverse events. The study design (S) covered any clinical trial, case report, case series, systematic review, meta‐analysis, umbrella review, or narrative review.

The inclusion criteria were as follows: (1) Studies that included pediatric patients (aged 0–18 years) diagnosed with COVID‐19 and developed oral manifestations. (2) Studies that evaluated oral manifestations or management strategies for oral manifestations in pediatric patients with COVID‐19. (3) Studies published in English. The exclusion criteria were: (1) Studies that did not report oral manifestations of COVID‐19 in pediatric patients. (2) Studies that did not report on management strategies of oral manifestations of COVID‐19 in pediatric patients. (3) Studies that were not published in English. (4) Commentaries and opinions were excluded from this review.

### 2.3 Search strategy

A search strategy was developed to identify relevant studies from electronic databases, including MEDLINE, Embase, Scopus, and Web of Science. The gray literature was also consulted through Google Scholar and OpenGrey. The MEDLINE database's search strategy was first defined via Pubmed using a controlled vocabulary (MeSH terms) and free keywords. The following search terms were used in the search: ((COVID‐19 OR coronavirus OR SARS‐CoV‐2) AND (oral manifestation OR oral lesion OR oral symptom OR mouth symptom OR oral cavity OR stomatitis)) AND (pediatric OR pediatric OR child OR children OR adolescent) AND (management OR treatment OR therapy OR intervention OR drug). Also done was a manual search of the reference lists of indicated research and pertinent review papers; from their beginning until the present, electronic databases have been analyzed. To be alerted of any new relevant research, a search alert was set for each database using its appropriate search method, and alerts were maintained periodically until January 2023. The search approach was meant to be exhaustive, sensitive, and particular to incorporate all relevant research.

### 2.4 Study selection and data extraction

All studies collected from the electronic search and other sources were uploaded to a reference management system, and duplicates were eliminated. Both the title/abstract screening, as well as the full‐text screening were carried out by two independent reviewers, identified by the initials K.N. and S.T. Cohen's kappa value was .85, indicating a high agreement level between the two reviewers. They independently screened the titles and abstracts of the studies retrieved from the databases according to the identical inclusion and exclusion criteria. The full text of the studies that met the initial screening criteria was then independently reviewed by them. Any disagreements between the reviewers were resolved through discussion or consultation with a third reviewer, identified by the initials M.B. A PRISMA flowchart was used to indicate the number of studies identified, screened, evaluated for eligibility, and included in the final evaluation. Included in the retrieved data were research information (author, publication year, study design, and sample size), participant characteristics (age, gender, and underlying medical problems), intervention or therapy, outcome measures, and outcomes. The procedure ensured the transparency, reproducibility, and precision of study selection.

### 2.5 Data analysis

Due to the variability of the included studies in terms of research design, demographic, intervention, and results, qualitative data synthesis was performed. The studies were evaluated based on the reported therapeutic techniques and their efficacy in treating oral symptoms of COVID‐19 in pediatric patients. The findings were summed together and presented in narrative format.

## 3 RESULTS

### 3.1 Studies selection

**Figure 1:**
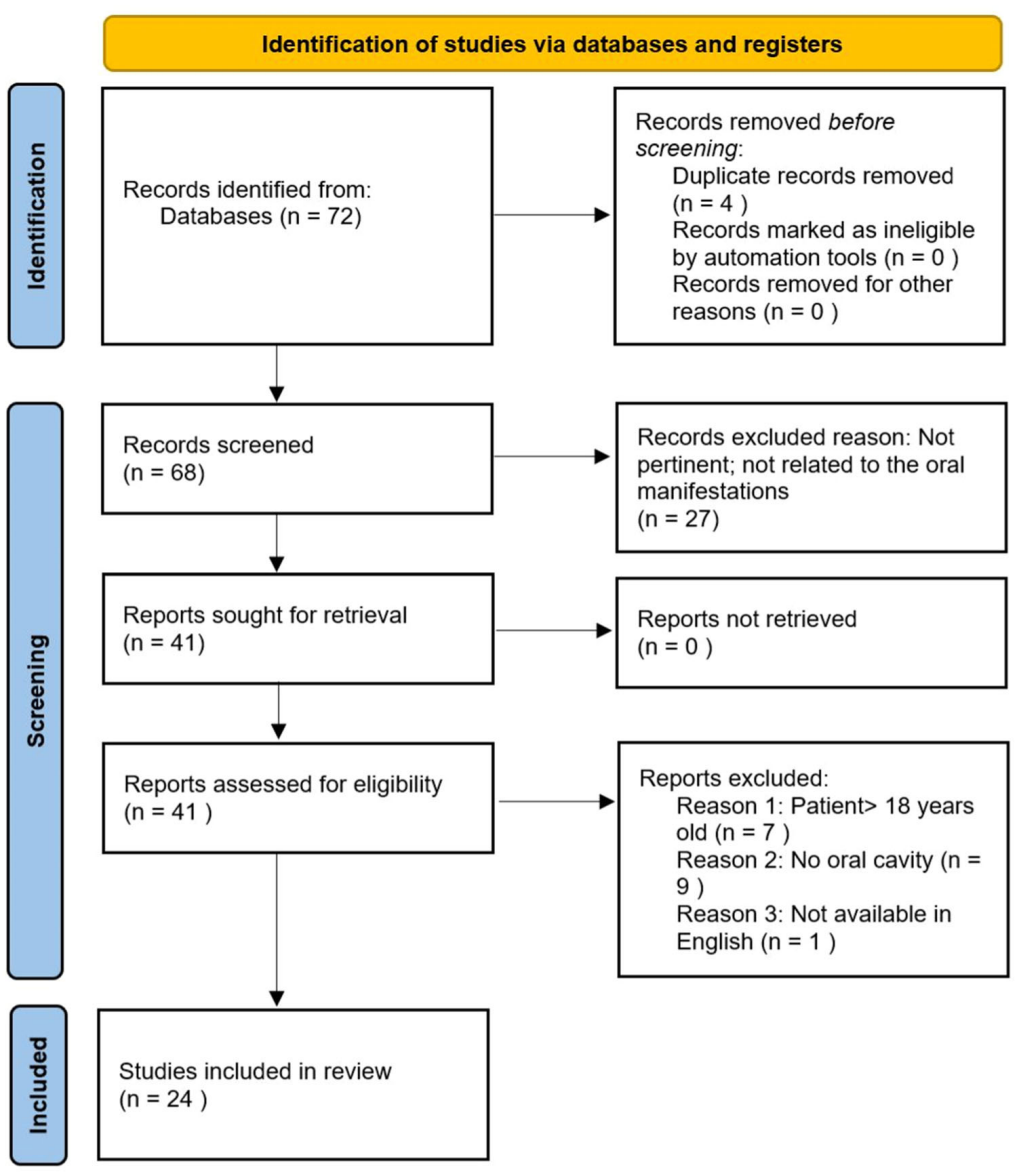
PRISMA flowchart for screened records.

The first search yielded 72 results. After deleting duplicates and screening titles and abstracts, the eligibility of 41 full‐text publications was evaluated. Twenty‐four of them matched the inclusion criteria and were included in the review. Figure 1 is a flowchart depicting the process of selecting studies from electronically received information.

### 3.2 Studies characteristics

The systematic review comprised 24 articles, nine of which were case reports, six were case series, one was a cross‐sectional study, four were reviews, two were retrospective, and two were cohort studies. Numerous countries, including the United States, China, France, Germany, the UK, Romania, Saudi Arabia, Italy, Brazil, and Iran, undertook the study. The sample sizes varied from 1 to 662 cases, and the review covered 2112 pediatric patients. The age range of the patients varied between research, with some involving just newborns and toddlers and others encompassing teenagers as old as 18 years. The majority of research were done in hospitals, while the rest were conducted in dentistry clinics or pediatric outpatient departments. The features of the included studies are displayed in Table 1.

**Table 1:** Characteristics of studies that meet the eligibility criteria included in the systematic review.

Study	Population	Study design	Oral manifestation	Limitations
Qiu et al. (2020)	10 adolescent patients with a range of 15–17 years	Case series	Gustatory dysfunction, hypogeusia	Differences in data collection methods and subjective perception of olfactory function are confounded
Mak et al. (2020)	3 patients with ages (14, 15, and 17 years)	Case report	Gustatory dysfunction, ageusia	Limited data
Ahmed et al. (2020)	662 patients (39 studies) with mean age 9.3	Systematic review	Cheilitis and tongue swelling	The level of evidence is low, and the risk of bias varies among studies
Jones et al. (2020)	1 patient (6 months)	Case report	Cracked lip and pominent papilla in tongue	‐
Rekhtman et al. (2021)	31 patients with a range of 1.75–16 years	Cohort	Lip fissuring and tongue papillitis	Small sample sizes
Cant et al. (2020)	1 patient (9 years)	Case report	Erosion and ulcers	‐
Dima et al. (2020)	3 patients with a range of 10–15 day	Case series	Oral candidiasis	Small sample size
Chiotos et al. (2020)	5 patients with a range of 5–14 years	Case series	Fissured lip	‐
Pouletty et al. (2020)	3 patients with a range of 4/7 to 12/5 years	Cohort	Cracked lip	‐
Labé et al. (2020)	2 patients with a range of 3–6 years	Case series	Erosion and hemorrhagic crusts in lips	‐
Mazzotta (2020)	1 patient (9 years)	Case report	Glossitis and cheilitis	‐
Chiu et al. (2020)	1 patient (10 years)	Case report	Cracked and erythema on lip and oropharynx	‐
Whittaker et al. (2020)	58 patients with a median of 9 years	Case series	Mucous membrane changes, and red cracked lips	It was based on retrospective data collection
Aghazadeh et al. (2020)	1 patient (9 years)	Case report	Vesicles and erosions	‐
Akca et al. (2020)	4 patients with a range of 8.5–10 years	Case series	Erosion and ulcers, maculae and petechiae	Small sample size
Al Ameer et al. (2020)	1 patient (13 years)	Case report	Maculae and petechiae, erythematous cracked lips	‐
Halepas et al. (2021)	47 patients with a range of 1.3–20.0 years	Cross‐sectional	Red or swollen lips, a strawberry tongue	‐
Bhujel et al. (2021)	84 patients (3 studies reported pediatric patients) with a range of 3 months to 20 years	Systematic review	Oral pseudomembranous candidiasis, geographic tongue, coated tongue, red or swollen lips, strawberry tongue	Most of the studies appraised had a high risk of bias
Naka et al. (2021)	452 patients (8 case series) with a median of 8–12 years	Review article	Dry and red lips and/or other mucosal changes	‐
Bardellini (2021)	27 patients with a range of 3 months to 14 years	Retrospective	Pseudomembranous candidiasis, geographic tongue, coated tongue, hyperemic pharynx	Retrospective design, limited generalizability
Brehm et al. (2022)	1 patient (10 Weeks)	Case report	Parotitis	‐
Likitnukul (2022)	1 patient (4 years)	Case report	Parotitis	‐
Santos et al. (2022)	89 patients with a range of 1–12 years	Retrospective	Mucositis, aphthous stomatitis, dentoalveolar abscess, hyperemic lips, exfoliative cheilitis, impetigo, and gingivitis	Reliability of the data collection, including assessment bias
Nascimento et al. (2022)	624 patients (25 studies) with mean age 8.78	Systematic review	Ulcers, dry and cracked lips, and strawberry tongue	The studies that were analyzed had a low to the moderated risk of bias

### 3.3 Synthesized findings

The studies found that oral manifestations of COVID‐19 in pediatric patients were less common than in adults. The most common oral manifestations reported in pediatric patients were mucosal lesions, including; petechiae, vesicular eruptions, erythematous macules, and ulcerations. Other reported manifestations include taste and smell disorders, oral candidiasis, hemorrhagic crust, tongue discoloration, lip and tongue fissuring, gingivitis, and salivary gland inflammation. The most often affected areas were the tongue, labial mucosa, palate, gingiva, buccal mucosa, and oropharynx, in that order (Aghazadeh et al., 2020; Ahmed et al., 2020; Akca et al., 2020; Bardellini, 2021; Bhujel et al., 2021; Cant et al., 2020; Chiotos et al., 2020; Chiu et al., 2020; Dima et al., 2020; Halepas et al., 2021; Jones et al., 2020; Kusiak et al., 2021; Labé et al., 2020; Mak et al., 2020; Mazzotta, 2020; Naka et al., 2021; Nascimento et al., 2022; Pouletty et al., 2020; Qiu et al., 2020; Rekhtman et al., 2021; Santos et al., 2022). Iatrogenic lesions (such as those caused by intubation and other invasive procedures), drug or vaccination reactions, dehydration, mouth breathing, a systemic inflammatory response or an altered immune system, and opportunistic co‐infections with *Candida albicans* or herpes simplex virus were all considered to be potential causes of oral manifestations other than SARS‐CoV‐2 infection. Multi‐system diseases characterized by a wide range of oral manifestations constituted the second group. Despite their widespread effect, oral symptoms are absent or merely present in a small percentage of people with clinical issues in this group. Diseases like Kawasaki‐like sickness and multi‐system inflammatory syndrome are included here. Mouth sores and involvement of the salivary glands are examples of the third group of oral symptoms caused directly by SARS‐CoV‐2 infection. Several reports have linked stomach issues, including nausea and vomiting, to oral cavity injury (Ahmed et al., 2020; Akca et al., 2020; Aragoneses et al., 2021; Di Spirito et al., 2022a; Halepas et al., 2021; Mak et al., 2020; Qiu et al., 2020; Rekhtman et al., 2021; Santos et al., 2022; Whittaker et al., 2020).

The incidence of oral symptoms in juvenile COVID‐19 patients varies significantly between studies, ranging from less than 1 percent to more than 60 percent (Aghazadeh et al., 2020; Ahmed et al., 2020; Akca et al., 2020; Bardellini, 2021; Bhujel et al., 2021; Cant et al., 2020; Chiotos et al., 2020; Chiu et al., 2020; Dima et al., 2020; Halepas et al., 2021; Jones et al., 2020; Kusiak et al., 2021; Labé et al., 2020; Mak et al., 2020; Mazzotta, 2020; Naka et al., 2021; Nascimento et al., 2022; Pouletty et al., 2020; Qiu et al., 2020; Rekhtman et al., 2021; Santos et al., 2022). There was no correlation between the intensity of COVID‐19 symptoms and the occurrence of oral manifestations. This shows that oral symptoms may be a specific feature of the therapy of COVID‐19 in young patients that is not well recognized. Further study is required to determine the precise processes behind the association between these oral symptoms and disease severity (Di Spirito et al., 2022a; Kumar et al., 2022). Young males (M:F = 20:15) may be more susceptible to oral lesions following viral infection, as evidenced by their higher incidence compared to females. This trend is also observed in adult subjects. This higher incidence in males could be attributed to severe forms of COVID‐19 requiring hospitalization, which is more prevalent among pediatric males (Di Spirito et al., 2022a, 2022b; Peckham et al., 2020). Managing oral manifestations of COVID‐19 in pediatric patients involves various measures, including preventive, therapeutic, and supportive care. Such a multifaceted approach includes preventive measures such as oral hygiene, regular oral care, and therapeutic measures such as medication. Despite the investigation, sufficient evidence‐based data specifically related to treating and managing oral manifestations of COVID‐19 in pediatric patients are not available yet.

Studies included in the systematic review ranged in quality. Due to their retrospective and anecdotal nature, case reports and case series constituted the vast majority of the research. Some studies lacked clear diagnostic criteria for COVID‐19, which could result in misdiagnosis or overdiagnosis of oral manifestations. Additionally, most studies did not report the severity or duration of the oral manifestations, making it difficult to assess their clinical significance. The bias risk assessment was not done systematically despite the authors' efforts due to the variety of studies. Overall, The evidence remains of low quality, and the danger of bias in the included research underscores the need for more high‐quality studies to strengthen the evidence base. The review's findings give valuable insights into the possible oral symptoms of COVID‐19 in pediatric patients and may help doctors manage these individuals.

## 4 DISCUSSION

This research aimed to provide a comprehensive analysis of the treatment options for children with oral manifestations of COVID‐19. This review considers articles that describe the oral signs of COVID‐19 in children and adolescents, as well as possible causes and treatments. This study uncovered new information on oral problems experienced by pediatric patients receiving COVID‐19 treatment. According to these data, oral symptoms are an underexplored aspect of COVID‐19 treatment in pediatric patients. Oral symptoms in young people infected with COVID‐19 are uncommon, and young men are disproportionately affected. The most common oral symptoms of pediatric COVID‐19 patients are oral candidiasis, mouth ulcers, and changes in taste and smell. The intensity of COVID‐19 symptoms did not appear to correlate with the severity of oral symptoms (Aghazadeh et al., 2020; Ahmed et al., 2020; Akca et al., 2020; Bardellini, 2021; Bhujel et al., 2021; Cant et al., 2020; Chiotos et al., 2020; Chiu et al., 2020; Di Spirito et al., 2022a, 2022b; Dima et al., 2020; Halepas et al., 2021; Jones et al., 2020; Kusiak et al., 2021; Labé et al., 2020; Mak et al., 2020; Mazzotta, 2020; Naka et al., 2021; Nascimento et al., 2022; Peckham et al., 2020; Pouletty et al., 2020; Qiu et al., 2020; Rekhtman et al., 2021; Santos et al., 2022). Several putative mechanisms, including direct viral infection, immune‐mediated inflammatory responses, and adverse medication reactions, have been hypothesized to explain the oral symptoms of COVID‐19 in pediatric patients. The incidence, severity, long‐term implications, and precise processes behind these oral symptoms require more study (Di Spirito et al., 2022a).

Preventing Oral COVID‐19 Symptoms in Children: Preventing the spread of COVID‐19 is similar to relieving the children's oral symptoms of the virus. Good hand hygiene, mask use, and personal space are all ways to lessen the spread of disease. Vaccination is another effective strategy for stopping the spread of COVID‐19 (Al‐Halabi et al., 2020; Banakar et al., 2020). Diagnosing oral manifestations of COVID‐19 in pediatric patients can be challenging, as these manifestations can also occur due to other medical conditions. When evaluating pediatric patients for oral manifestations of COVID‐19, healthcare providers must consider the differential diagnosis. This involves ruling out other possible causes of oral symptoms, such as infections (bacterial, viral, or fungal), autoimmune diseases, and allergies. A thorough medical and dental history, along with a physical examination, can help in the diagnosis. Providers may need to perform additional diagnostic tests such as imaging studies, laboratory tests, and biopsies to confirm the diagnosis and rule out other potential causes. In some cases, consultation with a pediatric dentist or specialist may be necessary to assess and manage these manifestations of COVID‐19 accurately. To effectively identify oral manifestations of COVID‐19 in pediatric patients, healthcare practitioners must consider several variables that may contribute to the symptoms. These characteristics include the patient's age, gender, and COVID‐19 infection severity. Oral lesions can be caused by several factors, including but not limited to improper dental care, opportunistic infections, stress, immunosuppression, vasculitis, and hyperinflammatory responses (Aragoneses et al., 2021; Di Spirito et al., 2022a; Iranmanesh et al., 2021; Swain et al., 2021). Clinicians should take a multidisciplinary strategy for treating oral symptoms of COVID‐19 in juvenile patients, involving pediatric dentists and oral medicine experts. In pediatric patients, treating oral symptoms of COVID‐19 is essentially supportive. Pain alleviation, oral hygiene measures, topical corticosteroids, antiviral medication, and chlorhexidine mouthwash may be used as treatment. Antiviral drugs may be administered in extreme situations (Brandini et al., 2021; Holt, 2021; Iranmanesh et al., 2021; Samuel et al., 2021).

### 4.1 Oral ulcers

Juvenile patients with COVID‐19 were most often diagnosed with oral ulcers, also known as canker sores or aphthous ulcers. These lesions may appear as ulcers, vesicles, or erythematous macules on the tongue, lips, buccal mucosa, and palate. Ulcers are typically small and painful sores on the tongue, gums, or within the cheeks. Several studies have found a correlation between COVID‐19 and mouth ulcers in young children (Akca et al., 2020; Cant et al., 2020; Di Spirito et al., 2022a; Labé et al., 2020; Nascimento et al., 2022). Little is known about what causes oral ulcers in young people with COVID‐19 infection. Oral mucosa direct infection by the virus leading to lesions has been postulated. A systemic inflammatory reaction to the illness is another possible cause of oral ulcers. More study is needed to determine what causes oral lesions in young people with COVID‐19. The most important risk factors for oral lesions in COVID‐19 patients are poor oral hygiene, opportunistic infections, stress, immunosuppression, vasculitis, and a hyperinflammatory response (Amorim dos Santos et al., 2020; Brandini et al., 2021).

Preventing oral lesions in pediatric patients includes good oral hygiene practices, such as regular brushing and flossing, and avoiding known irritants like tobacco and alcohol. Vaccination against infectious diseases, such as herpes simplex virus and human papillomavirus, can also reduce the risk of developing oral lesions. The management of oral lesions in pediatric patients depends on the underlying etiology and severity. The management is mainly symptomatic. Analgesics, such as acetaminophen or ibuprofen, are commonly used to manage pain associated with oral ulcerations. Topical anesthetics, such as benzocaine or lidocaine, can temporarily relieve pain. In cases where oral lesions are severe or interfere with oral intake, topical corticosteroids can be used. Antimicrobial agents, such as mouthwash or topical antibiotics, can also prevent secondary bacterial infections. Antiviral agents may be used for viral infections. In cases of bacterial infections, antibiotics are often indicated. In neoplastic processes, an appropriate referral to a specialist is necessary (Amorim dos Santos et al., 2020; Brandini et al., 2021).

### 4.2 Oral candidiasis

Oral candidiasis, often known as thrush, affects the mouth, throat, and tongue. While this illness is already frequent in children, there have been instances of thrush in Covid‐19‐positive pediatric patients (Bardellini, 2021; Bhujel et al., 2021; Dima et al., 2020). Oral candidiasis is caused by an overgrowth of candida fungus in the mouth. Candida is generally present in the mouth, but an overgrowth can emerge when the immune system is compromised. Additionally, Covid‐19 therapy may need antibiotics or corticosteroids, which are usually related to the development of oral candidiasis. It is hypothesized that people with Covid‐19 have a weaker immune system due to the virus. The majority of cases of oral candidiasis in children with covid‐19 were hospitalized and treated with a variety of medications, including broad‐spectrum antibiotics, most commonly azithromycin, corticosteroids, hydroxychloroquine sulfate, and Vitamin D, most commonly for pediatric cases, according to a case report (Bardellini, 2021; Bhujel et al., 2021; Dima et al., 2020).

It is important to seek medical attention for pediatric patients with covid‐19 presenting symptoms of oral candidiasis. Treating the underlying covid‐19 infection, treatment for oral candidiasis may include antifungal medication, such as nystatin, fluconazole, and itraconazole. It is important to complete the full course of antifungal medication as the doctor prescribes. Other measures to prevent the recurrence of oral candidiasis may include maintaining good oral hygiene, avoiding foods and beverages that can irritate the mouth, and using a spacer with inhaled corticosteroids to reduce the risk of developing thrush (Bardellini, 2021; Bhujel et al., 2021; Brandini et al., 2021).

### 4.3 Change of taste and smell

Loss of smell and taste are referred to, respectively, as anosmia and ageusia. Loss of taste and smell has been reported in children with COVID‐19, albeit this is far less common than in adults. This might cause a loss of appetite, leading to undernourishment and weight loss. There is a wide discrepancy in the studies' estimates of how often children with COVID‐19 lose their sense of taste and smell. Changes in study populations, testing methods, and the degree of COVID‐19 illness may all contribute to discrepancies in reported prevalence. Loss of taste and smell is one of the earliest signs of COVID‐19 in children and commonly occurs in tandem with other respiratory symptoms such as cough, fever, and sore throat. Partial or complete loss of taste and smell may persist for weeks or months after the onset of other symptoms has subsided. Some studies have suggested that older children and teenagers may be more at risk for losing their sense of taste and smell than younger children, but these results have not been replicated (Bodnia & Katzenstein, 2020; Hannum, 2021; Mak et al., 2020; Púa Torrejón et al., 2022; Qiu et al., 2020).

The precise mechanism underlying taste and smell disturbances in pediatric COVID‐19 patients is unknown. It is believed that the virus directly infects the cells that support and convey signals from taste and smell receptors, such as the olfactory epithelium in the nose, in cases of taste and smell problems. This can result in inflammation and cell damage, interfering with the normal transmission of taste and smell information to the brain. It is also likely that taste and olfactory problems in COVID‐19 patients are caused by general inflammation and immune system activation in the body, which can impact the function of the neurological system and sense receptors. However, further study is required to completely comprehend the processes causing this illness (Erdede et al., 2020; Mak et al., 2020; Parisi et al., 2022; Tsuchiya, 2021).

Children with COVID‐19 who lose taste or smell have few treatment options. However, symptoms can be reduced, and recovery is aided by receiving supportive care. Weight loss and loss of appetite are two side effects of losing the ability to taste and smell. Therefore, it's crucial to encourage healthy eating and drinking. Nasal irrigation with saline solution may also be beneficial, as it can help restore the sense of smell by reducing inflammation and clearing mucus from the nasal passages. In addition, some studies have suggested that olfactory training may be helpful for patients with persistent loss of smell. Olfactory training involves repeatedly exposing the patient to four distinct odors and asking them to identify each odor. This can help retrain the brain to recognize and respond to different smells. However, more research is needed to confirm the effectiveness of olfactory training in pediatric patients with COVID‐19 (Bodnia & Katzenstein, 2020; Erdede et al., 2020; Hannum, 2021; Púa Torrejón et al., 2022).

### 4.4 Salivary gland inflammation

COVID‐19 can cause salivary gland inflammation or sialadenitis in children and pediatric patients. The growth can cause discomfort and swelling in the mouth and may impair eating and speaking abilities. Parotitis is an inflammation of the parotid gland. Measles, influenza, adenoviruses, herpes simplex virus, and parvovirus B‐19 can all cause acute parotitis as a secondary viral infection. Little is known about the incidence of enlarged salivary glands in children with COVID‐19 (Brehm et al., 2022; Likitnukul, 2022; Moca et al., 2023). The SARS‐CoV‐2 virus might be transmitted from the salivary glands to the respiratory or digestive systems. If oral cells were better understood, it may help prevent the spread of viruses both within and outside the body. The fact that SARS‐CoV‐2 may be stored in the salivary glands provides a pathophysiological setting for studies that highlight the diagnostic potential of saliva for COVID‐19 and highlight the biological fluid's role in the spread of the disease (MohammadSadeghi et al., 2020; Pisano et al., 2023).

How COVID‐19 induces salivary gland enlargement in juvenile patients is not completely known. However, it is suspected that the virus either directly infects the salivary gland tissues or triggers an inflammatory response that results in swelling. Depending on the severity of the problem, the treatment choices for salivary gland enlargement in children with COVID‐19 may vary. Sometimes, supportive treatment, such as hydration and pain control, may suffice. Hospitalization and antibiotics or surgical intervention may be necessary in more severe situations as treatment options (Brandini et al., 2021; Rusu et al., 2021; Tsuchiya, 2021).

### 4.5 Kawasaki‐like disease (KD)

Numerous childhood symptoms have been linked to COVID‐19. The mortality rate for children with moderate or asymptomatic COVID‐19 is rising because more are developing severe systemic inflammation with fever and organ involvement. Pediatric multi‐system inflammatory syndrome is the name for this condition in children (MIS‐C) (Esposito & Principi, 2021). Symptoms of MIS‐C may include gastrointestinal distress, conjunctivitis, vomiting, breathing problems, diarrhea, myocarditis, or neurological manifestations. Multiple studies place the annual incidence rate of MIS‐C in those younger than 21 at about 5.1 per million. Early diagnosis is crucial for successfully treating this disease (Feldstein et al., 2020; Payne et al., 2021). There has been some skepticism about the causal function of SARS‐CoV‐2 in MIS‐C because several symptoms of MIS‐C are similar to those of KD. The similarity between MIS‐C and KD may be seen in their comparable clinical features (Sancho‐Shimizu et al., 2021).

KD is the leading cause of idiopathic acquired heart disease in children under the age of five, making it the most prevalent form of childhood vasculitis. Kawasaki illness may be caused by pathogenic microorganisms, according to some research. Children have developed vasculitis similar to KD during the current COVID‐19 epidemic. KD is characterized by a high temperature lasting more than ten days, a rash, swelling and peeling skin on the hands and feet, swollen lymph nodes, and red or irritated eyes, lips, and tongue (Gkoutzourelas et al., 2020; Moreira, 2020). KD can cause red, cracked lips as a symptom, along with other signs such as a strawberry tongue and inflamed oral mucosa. Although oral manifestations are not the primary symptoms associated with COVID‐19, some patients, including children, have experienced symptoms like dry mouth, taste alterations, and oral ulcers. The connection between KD and COVID‐19 is still under investigation, and more studies are needed to establish a clear link between the oral manifestations of the two conditions (Ahmed et al., 2020; Akca et al., 2020; Chiu et al., 2020; Jones et al., 2020; Labé et al., 2020; Nascimento et al., 2022; Pouletty et al., 2020).

Early recognition and diagnosis of Kawasaki illness in children is critical for preventing long‐term consequences such as coronary artery aneurysms. The treatment for KD typically involves intravenous immunoglobulin (IVIG) and high‐dose aspirin. In cases where Kawasaki‐like symptoms are linked to COVID‐19, additional treatments, such as steroids or other immunomodulating agents, may be considered (Esmaeilzadeh et al., 2021; Viner & Whittaker, 2020). For oral manifestations specifically, supportive care, such as maintaining good oral hygiene, using mouthwashes, and applying topical treatments for pain relief, can help manage symptoms. It is crucial to closely monitor patients with KD and COVID‐19 and tailor the treatment plan based on their individual needs and the severity of their symptoms. Collaboration between pediatricians, cardiologists, and other specialists may be necessary to ensure comprehensive care and optimal outcomes (Brandini et al., 2021).

### 4.6 Proposed guideline

In the context of the COVID‐19 pandemic, healthcare professionals must be cognizant of the different oral symptoms that may appear in young patients. These symptoms can enhance patient outcomes and minimize the likelihood of problems if they are recognized and treated promptly. Understanding the probable causes of oral symptoms in young COVID‐19 patients might also influence treatment options and future research. Table 2 gives diagnostic and treatment recommendations for oral symptoms of covid‐19 in children.

**Table 2:** Guidelines for diagnosing and managing oral manifestations of COVID‐19 in children.

Step	Guideline
1.	**Early recognition of oral manifestations in pediatric patients**:
	Educate healthcare professionals and parents about the possible oral manifestations of coronavirus disease 2019 (COVID‐19) in children, such as erythematous and cracked lips, oral ulcers, and tongue inflammation. Encourage parents to monitor their children for oral symptoms if they suspect COVID‐19 infection or if the child has been in contact with an infected individual.
2.	**Taking a history related to oral lesions**:
	1. Onset and duration: When did the oral lesion(s) first appear? Have the lesions changed since they first appeared? 2. Location and distribution: Where are the lesions located in the mouth? Are they localized or widespread? 3. Symptoms: Are the lesions painful or itchy? Do they cause discomfort while eating, drinking, or speaking? 4. Associated systemic symptoms: Has the patient experienced fever, fatigue, or respiratory issues? Are there any other skin rashes or lesions elsewhere on the body? 5. Medical history: Has the patient been diagnosed with COVID‐19 or been in close contact with someone who has? Does the patient have any pre‐existing medical conditions, such as autoimmune disorders or immunodeficiencies? Is the patient taking any medications that may cause oral lesions as a side effect? 6. Dental history: Has the patient experienced similar oral lesions in the past? When was the patient's last dental visit, and were any concerns raised then? 7. Family history: Is there a family history of oral lesions or conditions that may predispose the patient to develop oral lesions? 8. Personal habits and lifestyle: Does the patient practice good oral hygiene? Does the patient smoke or use tobacco products? What is the patient's dietary intake, and are they eating a balanced diet?
3.	**Diagnosis and assessment**:
	Strict infection prevention and control measures should be taken to prevent the spread of COVID‐19 in healthcare settings. Healthcare workers should wear appropriate personal protective equipment (PPE) when examining children with suspected COVID‐19. Perform a thorough oral examination: A thorough oral examination should be performed to assess for any oral manifestations of COVID‐19. Gently examine the oral cavity, including lips, tongue, cheeks, and palate. These may include ulcers or blisters on the tongue or oral mucosa, redness or swelling of the gums, or a dry mouth. Perform laboratory testing if needed: Laboratory testing may be recommended to confirm a diagnosis of COVID‐19. This may include a PCR, rapid antigen, or serology tests. Record the appearance, location, and severity of oral symptoms. Document and photograph oral manifestations to facilitate accurate diagnosis and monitor treatment progress. Consider referral to a pediatric dentist or oral medicine specialist if necessary.
4.	**Consider referral to a specialist if needed**:
	Biopsy or referral to a pathologist or oral medicine specialist for further investigation. Consider referral to a pediatric dentist or oral medicine specialist if necessary.
5.	**Management of oral manifestations**:
	Encourage good oral hygiene practices, including regular toothbrushing with a soft‐bristled brush and age‐appropriate fluoride toothpaste. Recommend the use of alcohol‐free mouthwashes containing anti‐inflammatory and antiviral agents, such as chlorhexidine or hydrogen peroxide, to reduce oral discomfort and prevent secondary infections. Prescribe topical anesthetics or analgesics for pain relief and inflammation control in case of oral ulcers or other painful oral manifestations. Instruct parents to keep their child well‐hydrated and to offer a soft, nonspicy, and nonacidic diet to minimize oral discomfort. Monitor for a referral to a pediatrician if symptoms worsen.
6.	**Management of associated systemic conditions**:
	Monitor pediatric patients with oral manifestations for signs and symptoms of the multi‐system inflammatory syndrome in children (MIS‐C) or Kawasaki disease. In cases of MIS‐C or Kawasaki disease, initiate appropriate treatment, including intravenous immunoglobulin (IVIG), high‐dose aspirin, and immunomodulating agents, under the guidance of a pediatric specialist or refer the patient to a pediatrician.
7.	**Follow‐up and monitoring**:
	Communication and collaboration between healthcare professionals, caregivers, and parents to ensure effective management of oral manifestations in pediatric patients with COVID‐19. Schedule regular follow‐up appointments to monitor the resolution of oral manifestations and to ensure appropriate management of any associated systemic conditions. Encourage parents to promptly report any new or worsening oral symptoms to their healthcare provider.
8.	**Further research and guideline development**:
	Encourage collaborative efforts among researchers, clinicians, and public health authorities to gather more data on the oral manifestations of COVID‐19 in children and their management. Continuously update and refine guidelines based on emerging evidence and best practices to ensure optimal patient care and outcomes.

### 4.7 limitations

The primary limitations of this systematic review are the small sample sizes, absence of control groups, and low‐quality assessment levels for the included studies. In addition, the research had varied study designs, treatments, and end measures, making it challenging to compare their results. To examine the prevalence, severity, and long‐term implications of oral manifestations of COVID‐19 in pediatric patients and to evaluate the efficacy of various oral care options, more research is required.

## 5 CONCLUSIONS

This systematic review provides a comprehensive analysis of the oral manifestations of COVID‐19 in pediatric patients and offers guidelines for their management. The results demonstrate that children with COVID‐19 can exhibit various oral symptoms, including oral lesions, taste and smell disorders, oral candidiasis, hemorrhagic crust, tongue discoloration, lip and tongue fissuring, gingivitis, and salivary gland inflammation. In children, the oral signs of COVID‐19 can either be the presenting symptom or arise later on throughout the illness. Therefore, healthcare providers must be aware of and consider these symptoms within the context of the current epidemic. Early identification of oral symptoms may lead to prompt diagnosis and management of COVID‐19, thus preventing potential complications and mitigating the spread of the virus. Management strategies should focus on alleviating symptoms, maintaining good oral hygiene, and addressing underlying causes or contributing factors. Additional treatments, such as topical or systemic medications, may sometimes be required. The exact pathophysiology of oral symptoms in juvenile COVID‐19 patients must be better understood to enhance diagnostic and treatment techniques. As the pandemic continues to evolve and new variants emerge, it is essential to remain vigilant for potential changes in the presentation and severity of oral manifestations in children affected by COVID‐19. Clinicians can provide optimal care by staying informed about the latest research and following evidence‐based guidelines.

## AUTHOR CONTRIBUTIONS

**Kamyar Nasiri**: Conceptualization, investigation, writing—review & editing. **Sahar Tehrani**: Investigation, visualization, validation. **Meysam Mohammadikhah**: Investigation, visualization, validation. **Morteza Banakar**: Conceptualization, supervision, project administration, writing—original draft, writing—review & editing. **Mojgan Alaeddini**: Investigation, writing—review & editing, data curation. **Shahroo Etemad‐Moghadam**: Investigation, validation, visualization, writing—review & editing. **Gustavo Vicentis Oliveira Fernandes**: Investigation, data curation, writing—review & editing. **Artak Heboyan**: Investigation, visualization, writing—review & editing. **Shima Imannezhad**: Conceptualization, supervision, writing—review & editing, project administration. **Farid Abbasi**: Conceptualization, methodology, writing—original draft.

## CONFLICT OF INTEREST STATEMENT

The authors declare no conflict of interest.

## Data Availability

The datasets used and/or analyzed during the current study are available from the corresponding author upon reasonable request.
